# Exacerbation of Evans syndrome in vaccinated pregnant woman with mild COVID-19 infection

**DOI:** 10.1016/j.xagr.2025.100574

**Published:** 2025-10-05

**Authors:** Vesna Elveđi-Gašparović, Dražen Pulanić, Petrana Beljan Džubur, Iva Miličić Pašalić, Ivo Vukasović

**Affiliations:** 1 Department of Gynaecology and Obstetrics, University Hospital Centre Zagreb, Zagreb, Croatia (Elveđi-Gašparović, Džubur, Pašalić, and Vukasović); 2 School of Medicine, University of Zagreb, Zagreb, Croatia (Elveđi-Gašparović, Pulanić and Džubur); 3 Division of Hematology, Department of Internal Medicine, University Hospital Centre Zagreb, Zagreb, Croatia (Pulanić)

## Introduction

Evans syndrome (ES) is a rare disease defined as concomitant or subsequent association of at least 2 of 3 autoimmune cytopenias: autoimmune hemolytic anemia (AIHA), immune thrombocytopenia (ITP), and autoimmune neutropenia. The course of the disease during pregnancy is usually benign. However, exacerbations can occur, posing significant management challenges. We report a case of a 30-year-old primigravida patient with a history of ES in remission who experienced severe exacerbation of thrombocytopenia at 20 weeks of gestation after a mild case of COVID-19, despite previous vaccination against SARS-CoV-2. Treatment with corticosteroids and intravenous immunoglobulin (IVIG) was initiated, resulting in partial improvement of platelet counts. In view of preterm premature rupture of membranes (PPROM), breech presentation, and severe maternal thrombocytopenia, cesarean delivery was performed at 35 weeks of gestation. This case highlights that COVID-19, even when mild and in vaccinated pregnant women, may trigger exacerbation of ES, complicating pregnancy management. The postpartum course was favorable. However, because of the lack of specific guidelines, close antenatal and postnatal multidisciplinary monitoring is required, and individualized care is essential to optimize both maternal and fetal outcomes. Our case contributes to the limited body of evidence by demonstrating that previous vaccination does not preclude disease flare, even in the context of mild infection.

In 1951, Robert Evans described a rare chronic autoimmune disease defined as a combination of ITP and AIHA with a positive direct antiglobulin test (DAT).^1^ The disease has a heterogeneous course, marked by chronic relapses.^2^ The exact pathophysiology remains unknown, although the trigger for the onset of the disease lies in the disorder of immune regulation.^3^^,^^4^ In pregnancy, the disease often follows a more benign course that responds to conventional therapy and frequently resolves after delivery.^5^ Furthermore, the treatment is especially challenging because of its potential teratogenic effects (eg, increased risk of spontaneous miscarriage; congenital heart disease; malformations of the limbs, trachea, and esophagus; nervous system malformations, such as spina bifida; etc.), in addition to the passive transplacental immune transfer of maternal immunoglobulin G (IgG) antibodies into the fetal circulation.^3^^,^^5^

## Case presentation

In May 2022, a 30-year-old primigravida patient was admitted to our department at 26 weeks of gestation because of the exacerbation of the patient’s underlying disease. The ES was diagnosed in early January 2017 with a predominant ITP with positive antiplatelet antibodies and a positive DAT showing anti-IgG positivity (1 + w). The patient was initially treated with corticosteroids and IVIG and then with 4 weekly doses of rituximab 375 mg/m^2^ as the second line of treatment. Because of ongoing severe ITP, therapeutic splenectomy was performed in late July 2017, achieving complete remission of the disease without the need for any concomitant medications. The patient was vaccinated against COVID-19 in March 2022.

In April 2022, at 20 weeks of gestation, the patient had suffered a mild course of COVID-19 (the patient presented with a cough, a sore throat, and fatigue), which was followed by a rapid decrease in platelet count (41 × 10^9^/L). Before the infection, platelet levels were 107 × 10^9^/L with normal red blood cell parameters. The initial phase of treatment was performed in an outpatient setting, between 20 and 26 weeks of gestation, including methylprednisolone (32 mg), which resulted in temporary and fast improvement of serum platelet count with tapering of corticosteroid therapy. At 26 weeks of gestation, on admission to our department of gynecology, the platelet count was 41 × 10^9^/L, but fetal sonography and other laboratory values were unremarkable. Since admission to our department at 26 weeks of gestation until delivery, we performed daily cardiotocography (CTG) monitoring. Fetal anatomy was normal, and growth assessment was within normal ranges. Doppler parameters were evaluated on a weekly basis and were normal. No sign of fetal intracranial hemorrhage was detected during the sonographic follow-up. The patient’s platelet counts decreased to 15 × 10^9^/L, and the patient started with higher corticosteroid doses (methylprednisolone 1 mg/kg). However, because of inadequate improvement of platelet count, IVIG was administered over a 3-day course (30 g per day), resulting in temporary improvement in platelet count.

This regimen was repeated on 3 separate occasions at 29, 32, and 34 weeks of gestation. After each administration, the platelet count remained above 40 × 10^9^/L for an average duration of approximately 3 weeks (Figure). At 35 weeks of gestation, PPROM occurred, and a cesarean delivery was performed because of an unfavorable cervix and footling breech presentation. Moreover, severe maternal thrombocytopenia posed a risk of fetal hemorrhage, considering the potential transplacental transfer of antiplatelet antibodies. A healthy male newborn weighing 2570 g and 46 cm long with Apgar scores of 10/10 was delivered. The placenta weighed 370 g with normal macroscopic appearance and histologic findings. There was no postpartum hemorrhage. IVIG (30 g) with methylprednisolone and 4 doses of platelets were administered preoperatively, alongside 4 doses of platelets intraoperatively. Platelet values were 23 × 10^9^/L preoperatively and 136 × 10^9^/L postoperatively. The maternal platelet count started to improve on the second postpartum day. The trend continued in the following days, leading to a platelet count of 126 × 10^9^/L on postpartum day 8 when the patient and baby were discharged from the clinic. The neonate tests for ITP and AIHA were negative. Neonate laboratory findings on the day of birth were a hemoglobin level of 164 × 10^12^/L and a platelet count of 315 × 10^9^/L. Lactation was established, and the patient continued breastfeeding throughout the postpartum period. The patient gradually tapered and finally stopped corticosteroid therapy several weeks after delivery and was in complete remission of ITP/ES for the next 3 years without the need for any treatment.

**Figure:**
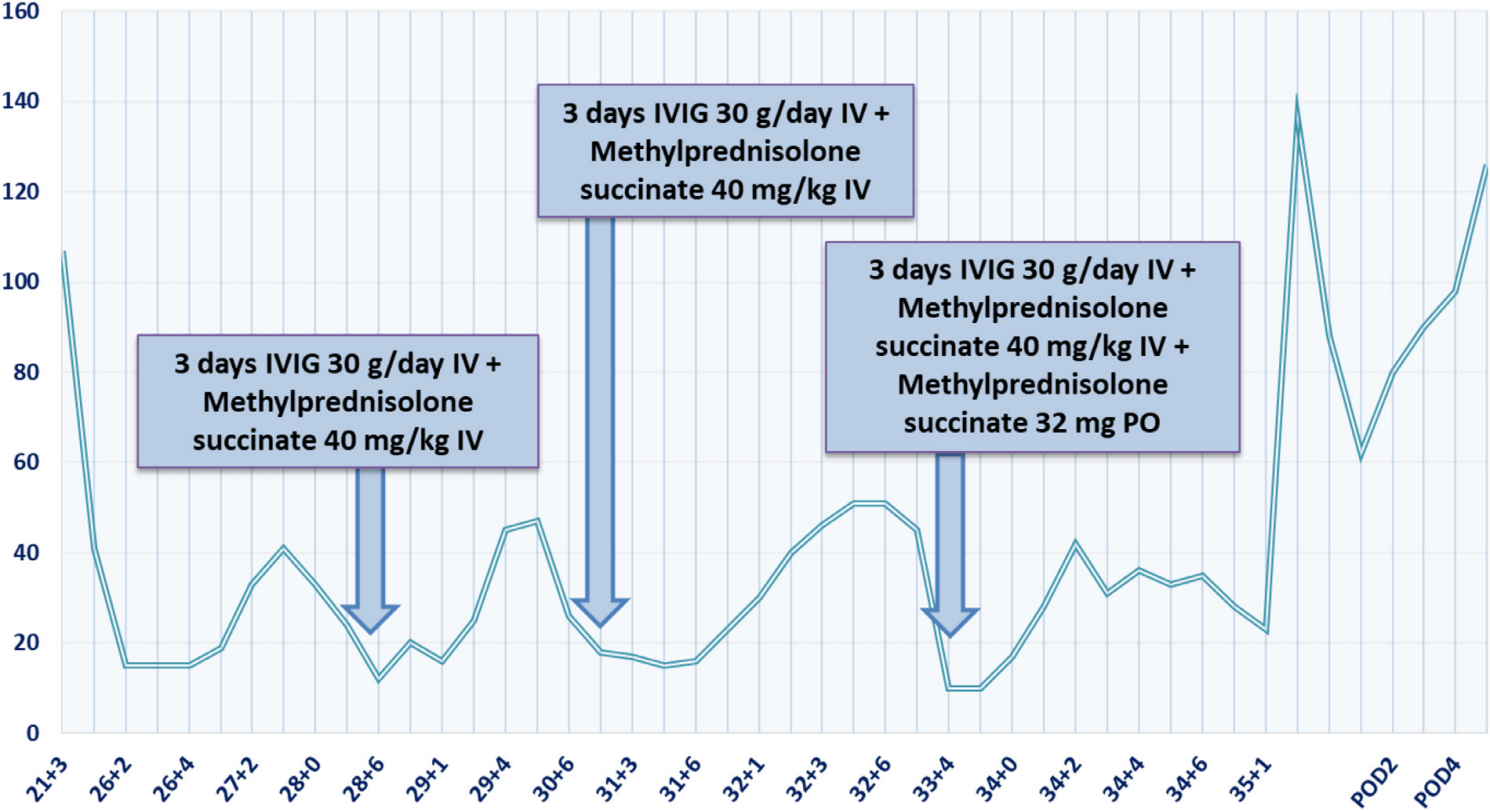
Platelet response to therapy during pregnancy *IV*, intravenous; *IVIG*, intravenous immunoglobulin; *POD*, postoperative day; *PO*, per os.

Elveđi-Gašparović. Worsening Evans syndrome in vaccinated pregnant woman with COVID-19. Am J Obstet Gynecol Glob Rep 2025.

## Discussion

ES is a rare disease defined as the concomitant or subsequent association of at least 2 of 3 autoimmune cytopenias (ITP, AIHA, and autoimmune neutropenia).^1^^,^^6^ The occurrence of ES during pregnancy has been rarely reported. Given its rarity, available data concerning the clinical course and optimal management in pregnancy are limited. The course of the disease during pregnancy is usually benign, with an increased risk of placental abruption and postpartum hemorrhage.^4^ Because of its typical remission-relapse course, ES may develop for the first time in pregnancy or recur in patients with a previous diagnosis. Preconceptional counseling for patients with ES is extremely important because such a condition carries risks for both the mother and fetus, requiring a multidisciplinary approach to ensure favorable perinatal outcomes. Counseling addresses the potential for severe pregnancy complications and fetal morbidity and mortality because of antibody transfer and guides maternal management and treatment options. Management depends on the severity of the cytopenia, but during pregnancy, it leads to significant challenges because of the lack of specific guidelines^7^^,^^8^ and concerns about potential adverse effects of medications. Close antenatal monitoring emphasizes an individualized treatment plan and coordinated team of specialists in hematology, obstetrics, and neonatology to improve the chances of a successful pregnancy outcome. Although the incidence of ES in pregnancy is not well known, it has been diagnosed in 1.8% to 10.0% of patients with ITP.^9^ In pregnant patients with anemia and thrombocytopenia, it is important to differentiate ES from causes that are more common in pregnancy. The differential diagnosis was particularly challenging in our case because it was difficult to determine whether the thrombocytopenia was due to an exacerbation of the underlying disease or a symptom of SARS-CoV-2 infection. A low platelet count is a common feature of COVID-19 and has been considered a poor prognostic factor.^10^ The effect of COVID-19 on the course of pregnancy remains unclear. In our case, the diagnosis of ES had been made before the pregnancy, and the patient had previously undergone several lines of treatment. Although the disease typically follows a mild course during pregnancy, our patient experienced an exacerbation. The likely trigger was COVID-19 that occurred at 20 weeks of gestation. Despite having a milder form of the infection and being vaccinated before the pregnancy, the patient experienced a significant drop in platelet count. In addition, we noted positive IgG antiplatelet antibodies. However, according to current guidelines, these findings have no diagnostic significance either during or outside of pregnancy, and they do not have a predictive role in neonatal thrombocytopenia.^11^ Guidelines regarding perinatal care are constantly being updated to balance evidence-based maternity care with COVID-19 management and treatment strategies. This case highlighted the influence of COVID-19 on the rapid deterioration of ES, making it a risk factor for an adverse course of pregnancy.

Therapeutic options in pregnancy are specifically challenging, as a large number of drugs cannot be administered because of the crossing of the fetoplacental barrier and the potential teratogenic effect on the fetus. Our patient was treated with corticosteroids and IVIG, achieving an increase in platelet count, which lasted 2 to 3 weeks, and maintaining a platelet count above 20 × 10⁹/L. There is no published comparative research yet on the use of corticosteroids and IVIG in pregnancy in this indication. Decisions regarding the mode of delivery and choice of medications had to be made on a case-by-case basis by an experienced multidisciplinary team. Previous research has not provided sufficient evidence to support clear recommendations or guidelines for determining the optimal method of birth in such cases. The primary and still unresolved challenge lies in assessing the risk of fetal thrombocytopenia and, consequently, the risk of neonatal hemorrhage. Therefore, the mode of delivery remains based solely on obstetrical indications.^11^ In our case, cesarean delivery was performed because of PPROM, extremely severe maternal thrombocytopenia, unfavorable obstetrical status, and footling breech presentation.

COVID-19 is known to be a trigger for various secondary autoimmune diseases, and only 2 case reports were available on ES and COVID-19 during pregnancy.^4^^,^^12^ There is a lack of data on the effect of COVID-19 on the course of pregnancy with previously known ES, and each such case is exceptionally helpful to clinicians. Therefore, a comparison of the course of the disease in vaccinated and unvaccinated pregnant women is not possible. In a recent systematic review of ES associated with COVID-19 or COVID-19 vaccination, Yacoub et al^13^ identified 11 cases of ES associated with COVID-19 and 5 cases of ES associated with COVID-19 vaccination. In that cohort, there was only 1 pregnant woman who developed ES during COVID-19. This pregnant woman was not vaccinated. In contrast to the Fayed and Santosa case presentations of secondary ES caused by severe coronavirus disease in nonvaccinated pregnant women, our presented case highlighted that, despite vaccination and a mild clinical course of COVID-19, the infection in pregnant women with ES can still trigger deterioration of the primary disease and significantly affect the course of pregnancy and perinatal outcome. Our case adds to this limited body of evidence that vaccination does not preclude disease flare, even with mild infection. Fontana et al^14^ have recently presented a case of a 32-year-old woman who experienced ES during her first pregnancy, with previously diagnosed childhood-onset systemic lupus erythematosus, highlighting a clinical challenge of the need for multiple treatments, including conventional immunosuppressants and/or biologic drugs as steroid-sparing agents. According to Jiang et al,^15^ outside of pregnancy, ES features higher mortality and disease-related complications than isolated ITP or AIHA. Currently, there is no antenatal measure that is able to predict the occurrence of ES-related cytopenia in fetuses, as this is not related to maternal blood cell count or maternal response to therapy.^16^ A close fetal ultrasound monitoring is necessary to detect early signs of cytopenia. Multidisciplinary approach and tertiary center care significantly contribute to a favorable perinatal outcome. Despite the latest recommendations,^6^ there is still no clear strategy for the therapeutic approach in pregnancy.

Because of the lack of specific guidelines, the presentation of each new case of a pregnant woman with ES will contribute to collecting as much data as possible to ensure optimal care, even in the most complex and specific situations.

## CRediT authorship contribution statement

**Vesna Elveđi-Gašparović:** Writing – review & editing, Supervision, Conceptualization. **Dražen Pulanić:** Writing – review & editing. **Petrana Beljan Džubur:** Writing – review & editing, Writing – original draft. **Iva Miličić Pašalić:** Writing – original draft. **Ivo Vukasović:** Writing – review & editing.
